# Extraction, Composition, Functionality, and Utilization of Brewer’s Spent Grain Protein in Food Formulations

**DOI:** 10.3390/foods12071543

**Published:** 2023-04-05

**Authors:** Bhanu Devnani, Galo Chuchuca Moran, Lutz Grossmann

**Affiliations:** Department of Food Science, University of Massachusetts Amherst, Amherst, MA 01003, USA; bdevnani@umass.edu (B.D.); gchuchucamor@umass.edu (G.C.M.)

**Keywords:** food waste, alternative proteins, cereal protein, sidestream, prolamins

## Abstract

In recent years, brewer’s spent grain (BSG) has gained attention as a plant-based protein source because it occurs in large quantities as a by-product of beer brewing. BSG can contribute to future food requirements and support the development of a circular economy. In light of the dynamic developments in this area, this review aims to understand the proteins present in BSG, and the effect of extraction techniques and conditions on the composition, physicochemical, and techno-functional properties of the obtained protein extracts. The water-insoluble hordeins and glutelins form the major protein fractions in BSG. Depending on the beer brewing process, the extraction technique, and conditions, the BSG protein isolates predominantly contain B, C, and ϒ hordeins, and exhibit a broad molecular weight distribution ranging between <5 kDa and >250 kDa. While the BSG isolates obtained through chemical extraction methods seem promising to obtain gelled food products, physical and enzymatic modifications of BSG proteins through ultrasound and proteolytic hydrolysis offer an effective way to produce soluble and functional protein isolates with good emulsifying and foaming capabilities. Specifically tailored protein extracts to suit different applications can thus be obtained from BSG, highlighting that it is a highly valuable protein source.

## 1. Introduction

The valorization of sidestreams from plant food processing plays a vital role in today’s day and age when plant proteins have gained considerable attention to foster the transition towards a more sustainable protein supply for the growing population. Brewer’s spent grain (BSG) is the sidestream that remains after the wort production during the beer brewing process. While the generated wort is utilized for the fermentation process, the BSG is not used any further in the brewing process. 

BSG accounts for around 85% of the total sidestreams generated by the brewing industry, with 20 kg of it being produced for every 100 L of beer [1]. Global beer production has been ranging between 1.9 and 2 billion hectoliters in the last decade [2], implying the generation of 38 to 40 million tons of BSG annually. Continuous efforts are being made by the food industry to expand the utilization of this sidestream, which currently is limited to animal feed, composting, and biogas production [3]. BSG can, however, act as an alternative protein source, owing to the presence of approximately 15–30% of protein, suggesting its potential to act as a suitable raw material for high-protein plant-based product formulations. In addition to protein, BSG is rich in dietary fiber and contains small amounts of lipids, starch, and ash (Table 1), and appreciable levels of vitamins, minerals, and polyphenols [4,5,6,7]. The high nutritional density, coupled with consistent year around availability, large production volumes, and low market price, make it an attractive raw material for human food applications. Although BSG has been used to enhance the overall protein content of staple human foods such as bread and pasta [8,9], the anti-nutrients present in it—particularly polyphenols [6]—may restrict the overall nutrient absorption and utilization [10]. Extracting proteins from BSG, therefore, seems like a reliable approach to enhancing the protein content and bioavailability, while offering greater flexibility in formulations.

The type, content, and properties of proteins present in barley, the principal ingredient used for beer production, are shown in Table 2. Prolamins and glutelins form the major protein fractions in the barley grain, constituting approximately 35–55% and 35–45% of the total protein, respectively, the rest being albumins and globulins [11]. Hordeins are the major form of the prolamin group of proteins and are further classified into B, C, D, ϒ, and other low molecular weight hordeins based on the presence of sulfur-containing amino acids and the electrophoretic mobility (Table 2) [12,13]. The C and D hordeins are S-poor (1–2%) and have higher MWs ranging from 55 to >100 kDa. The B and ϒ hordeins are high in S-containing amino acids (4–6%) but have lower MWs of ~35–50 kDa. The presence of proteins with even lower molecular weights of ~10–25 kDa, initially referred to as A-hordeins, have also been reported in early studies; these, however, were later found to be a mixture of some prolamin-like proteins and enzyme inhibitors rather than true prolamins [14,15]. Some hordeins, specifically B and D hordeins, also occur in the glutelin fraction [16]. The barley albumins mainly include protein Z and lipid transfer proteins (~80%). These are rich in the amino acid lysine (4–7% vs. 2% in the other protein fractions), the most common deficient amino acid in cereals. The hordeins, glutelins, and globulins in contrast are rich in S-containing amino acids, with these containing ~4–9% compared to only ~2% in albumins, except for lipid transfer proteins (Table 2).

**Figure 1:**
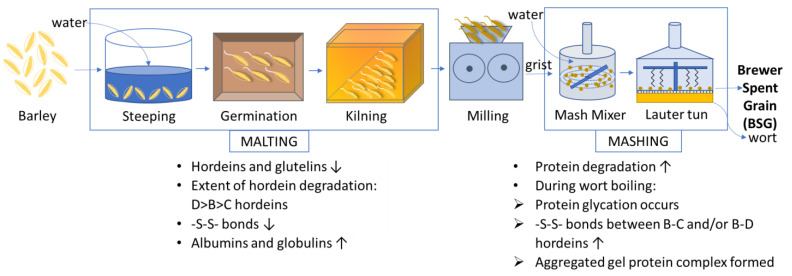
Protein compositional and structural changes during conversion of barley to BSG. Information adapted from: Osman et al., 2002 [25]; Marchylo et al., 1986 [29]; Tatham and Shewry, 1995 [30]; Wallace and Lance, 1988 [31]; Celus et al., 2006 [16]; Jones and Marinac, 2002 [28]; Steiner et al., 2011 [20]; Smith and Lister, 1983 [26]; Smith and Simpson, 1983 [27].

BSG is mainly comprised hordeins and glutelins (43% and 21.5% of the total extractable protein, respectively), with albumins and globulins only being present at low concentrations (7.5%) [4,16]. The structural changes that barley proteins undergo during malting and mashing have been discussed in detail in a recent review [11]; a summary of these changes is presented in Figure 1 to understand how the protein composition changes during processing. Briefly, during malting, the proteolytic enzymes hydrolyze more than 70% of the hordeins and glutelins with D hordeins showing the highest degree of hydrolysis, followed by B and C hordeins [11,25,26,27]. As a result, the disulfide crosslinks in hordeins are reduced by ~60%, leading to a change in protein classification because the hydrolysis induces a transition in solubility, with especially albumins and globulins being increased in the malt [16]. During mashing, these albumins and globulins solubilize in the water phase and the protein degradation continues until the mash is subjected to boiling [28]. Moreover, glycation reactions of soluble proteins occur during boiling and these glycosylated proteins then permeate into the wort [20]. In contrast, the insoluble proteins (mainly hordeins and glutelins) experience enhanced disulfide crosslinking and aggregation. The extensive crosslinking eventually results in the formation of a gelled complex, which settles at the bottom of the mash and forms the major protein component of BSG [16]. It is important to note that, while hordeins and glutelins form the major protein fractions of both barley and BSG (80% in total), their structural rearrangements during the brewing process are expected to influence their extractability in different solvents and their physicochemical properties.

The market for plant-based proteins has been very dynamic in recent years, with researchers and product developers constantly striving to discover novel, highly functional alternative protein sources. BSG has the potential to be used as a highly viable plant protein source providing possible economic, nutritional, and sustainability benefits [32,33]. The extraction technique and the processing parameters during extraction, however, influence the technological and physicochemical properties of proteins. While some extraction techniques used to isolate BSG proteins have been recently reviewed [11,32,34], a review focusing on the relationship between extraction techniques/conditions and the BSG protein structure as well as function is still needed to understand the opportunities and challenges existing in the commercial utilization of BSG. This knowledge is imperative to expand the use of BSG proteins in a variety of food and beverage applications ranging from protein powders to meat alternatives. Thus, through this review, we aim to fill this gap and provide the reader with comprehensive knowledge of the compositional, structural, functional, and nutritional properties of BSG proteins, as a function of extraction techniques and conditions.

## 2. Protein Extraction Approaches

As wet BSG obtained after the brewing process contains high amounts of moisture (75–80%); it is first dried to enhance the shelf life, reduce the volume, and ease material handling [35]. After drying, a variety of extraction methods and conditions have been reported to obtain protein products from BSG (Table 3), with alkaline extraction being the most commonly used approach [36,37,38,39,40]. Most extraction methods, in general, include two steps: protein solubilization followed by protein recovery. The BSG is first mixed with water-based solutions, which may contain a variety of reagents that help disintegrate the BSG matrix and decrease interactions between proteins and other macromolecules, thus facilitating their release. The solubilized proteins are then recovered either through centrifugation and isoelectric point precipitation [37,38,40,41,42] or membrane filtration [39,43,44,45].

This section reviews different extraction methods and the variations in the processing parameters in each of the extraction methods. Extraction yield and protein purity have been taken as measures to compare the effectiveness of various extraction procedures, with the former being defined as protein recovery based on BSG protein content, and the latter being the protein content in the isolated protein.

### 2.1. Pretreatments

A set of different pretreatments are often used before extraction, to assist protein release from the BSG matrix. Defatting, particle size reduction through milling or shearing, enzymatic hydrolyzation, and ultrasound are the most common pretreatments, with the latter two also being used independently as ‘chemical free’ alternatives to traditional alkaline extraction (Table 3) [37,38,39,40,41,42,44,47,48,54]. Mixed results, however, have been reported after studying the effects of these pretreatments. While Connolly et al. [37] achieved a threefold increase in extracted proteins after blending a BSG solution in a high-shear blender prior to alkaline extraction, Qin et al. [39] and Junttila [46], found no significant effects of grinding and defatting of the BSG before alkaline or acid extractions. Interestingly, Karlsen et al. [38] found that defatting the BSG decreased the extraction yield from 45 to 38% compared to raw BSG, potentially due to the solubilization of proteins in the methanol used during the defatting process. No significant differences were, however, reported between the protein purity of these extracts.

Additionally, it is important to note that differences in the raw materials and processing parameters during brewing might alter BSG composition and, consequently, the protein extraction. While this information is lacking in most of the studies in the literature, a study by Connolly et al. [37] highlighted the impact of the degree of roasting during brewing on protein extraction. Only 15% of the protein was recovered from BSG derived from dark malt (i.e., highly roasted malt) compared to 59% from pale BSG using alkaline extraction. More studies are needed in this area to specifically understand the effect of malt processing and brewing on the extractability of BSG proteins and their properties.

### 2.2. Alkaline Extraction

Alkaline extraction is a well-studied method to recover proteins from different kinds of agricultural sources, including BSG. In this method, the alkaline conditions facilitate protein solubilization by altering protein structure, charge, and, consequently, interactions [36,37,38,39,40,44,47,48,58]. A wide range of extraction yields (~18 to 82%) and protein purities (37 to 69%) have been obtained through this method upon varying the process conditions, as shown in Table 3.

The key variables determining the effectiveness of the extraction process include the type of alkali used as well as its concentration, the extraction temperature, the solid: liquid ratio, and the isoelectric point used for protein precipitation. While sodium hydroxide has been the most commonly employed alkali to obtain alkaline conditions during the production of BSG protein isolates, its concentration in the solution is known to have a profound influence on extraction yield and purity. For instance, a ~twofold increase in the extraction yield was achieved when the sodium hydroxide concentration was increased from 0.05 to 0.15 M and from 0.01 to 0.1 M [37,46]. Similar results were obtained by He et al. [44]. In this study, the protein recovery increased from 65 to 82% after increasing the sodium hydroxide concentration from 1 to 5%. The increase in alkali concentration, however, lowered the purity of the resulting protein extracts, potentially due to the increased solubilization of carbohydrates at such extreme pH conditions. On a side note, heating the BSG dispersion to 50 to 60 °C during extraction also led to a similar effect of higher solubilization of swollen starch granules bound to proteins, affecting protein purity [37,44].

Other extraction parameters have also been investigated and their influence on protein yield and purity was described:

- Changing the solid-to-liquid ratio resulted in a maximum extraction yield of proteins at a ratio of 1:20 (4.8% *w*/*v*) [37].
- Researchers utilized a wide range of pH values ranging from pH 2 to 4.5 to precipitate the proteins. This is due to the presence of different proteins found in BSG that are known to have isoelectric points ranging from pH 6 (hordeins) to around pH 3.7–4.5 (glutelins) [59,60].
- Increasing the number of extraction cycles also influenced the protein yield. Qin et al. [39], for instance, obtained a higher extraction yield of 78% after two extraction cycles, compared to most of the studies (Table 3). Similarly, Karlsen et al. [38], improved the extraction yield from 25 to 45% after three extraction cycles. Nonetheless, while multiple extraction cycles enhanced the extraction yield, the protein purities of the extracts remained low (42%).

The consistently low protein purities of 37 to 69% across various studies indicates that efficient fractionation procedures are required to separate proteins from the high amounts of fiber present in BSG to obtain a high protein purity. Future work in this direction, coupled with efforts to reduce the use of alkaline reagents and water volume, is needed to extract proteins sustainably.

### 2.3. Acid, Reducing Agent, and Salt Extraction

BSG is a complex food matrix that contains proteins entrapped within complex carbohydrate structures. Extraction methods using acids and reducing agents, such as sulfuric acid and sodium bisulfite, aim to hydrolyze the hemicellulose present in the cell walls of raw materials, and break the disulfide bonds in proteins, increasing their solubility and facilitating their recovery [44]. Moreover, the proteins are partly hydrolyzed in this procedure, which increases their solubility [48]. Higher extraction yields of ~63 to 90%, but lower protein purities of ~24 to 39% were achieved using this method compared to alkaline extraction (Table 3). This suggests simultaneously enhanced recovery of both proteins and carbohydrates. In addition to acids, autoclaving at temperatures of 121 to 130 °C for a duration of ~30 to 60 min was also used in these methods to catalyze the hydrolysis of the cell wall structures [39,46,49].

Aiming to determine the optimal processing conditions, different concentrations of acids and reducing agents have been evaluated. Qin et al. [39] reported that increasing the sulfuric acid concentrations from 2 to 4% recovered more than 85% of the proteins in BSG and reduced by 50% the autoclaving time. However, no significant differences in protein separation efficiencies were observed by He et al. [44] upon varying the sodium bisulfite concentration from 1 to 5% *w*/*w* of dry BSG. This was attributed to the inability of reducing agents to dissociate the proteins from the cellulosic material present in BSG. The study recommended that the addition of detergents such as sodium dodecyl sulfate (SDS) during the extraction could enhance the protein solubility. However, when utilizing acidification and reducing agents it has to be kept in mind that these compounds need to fulfill all regulatory aspects applicable to food use.

Salt extraction combined with surfactants, using SDS and disodium phosphate (DSP), promotes the solubilization of the protein aggregates of BSG [50]. Extraction yields of 50 to 60% were achieved using SDS concentrations from 0.5 to 3% and 0.5% of DSP (Table 3). During the extraction, the BSG and solvent mixtures were heated up to ~100 °C for a duration of 60 to 120 min [50,51]. At a set duration of 60 min, an increase in the extraction yield from 17 to 49% was observed upon increasing the extraction temperature from 27 to 100 °C, perhaps due to enhanced protein solubilization [50]. Diptee et al. [51], in addition, studied the effect of the salt concentration and concluded that higher concentrations of SDS interestingly do not increase protein recovery of BSG. In both studies, 70 to 95% of ethanol solution at 4 °C was used to precipitate proteins as it helps to reduce the dielectric constant and facilitates separation by enhancing protein−protein interactions [50,51].

### 2.4. Solvent Extraction

The two solvent extraction techniques that have been evaluated to isolate proteins from BSG include the use of deep eutectic solvents and pressurized solvent extraction. Deep eutectic solvents can fractionate and dissolve lignin and starch compounds in the raw material, facilitating the release of proteins [45]. High temperatures and pressures during pressurized solvent extraction enhance solvent penetration into the sample matrix, requiring lower amounts of chemicals and resulting in higher extraction yields [41]. To accelerate the extraction in both methods, the prepared BSG−solvent mixture is heated at high temperatures (150 °C) for short periods (10 min) or at mild temperatures (80 °C) for long periods (4 h) during pressurized solvent extraction and deep eutectic solvent extraction, respectively. The proteins are then recovered in the liquid fraction. Interesting insights have recently been reported for both extraction methods.

First, after evaluating different deep eutectic solvents, Wahlström et al. [45] found that a combination of sodium acetate with urea (1:2) at 80 °C for 4 h was able to extract 79% of proteins from BSG. A similar yield (78%) was reached for a mixture of potassium acetate and urea (1:3). Although the extraction yield was high, isoelectric point precipitation of the proteins from the liquid extract was not feasible due to the buffering effect of the salts. Dialysis was proposed to lower the ion concentration, but it may limit the scalability from an industrial point of view. Second, González-García et al. [41] evaluated the use of pressurized solvent extraction to recover BSG protein and demonstrated that higher temperatures (155 °C) promoted the extraction of the proteins compared to lower temperatures (25 and 90 °C). A maximum of 69% of the proteins could be extracted from BSG at the optimized conditions utilizing 4.7% ethanol at 155 °C for 10 h during a five-cycle extraction procedure. This extract, however, had a low protein purity of 20%.

In the terms of industrial scalability, pressurized solvent extraction in a biorefinery is feasible when considering the process itself, the equipment needed, and the costs required [61]. The use of solvents and high temperatures during the extraction, however, could alter the functional properties of BSG proteins. More studies focusing on the effect of extraction conditions on the structure and functional properties of proteins would offer a more comprehensive conclusion. Moreover, toxicological studies and discussion on the regulatory aspects are needed for both extraction methods.

### 2.5. Hydrothermal and Subcritical Water Extractions

Hydrothermal extraction uses water as a solvent at mild to high temperatures for short or long periods to facilitate the release of proteins [52]. Subcritical extraction also uses water but at temperatures higher than 100 °C, where its liquid state is maintained using a pressurized system. Under these conditions, water has a higher ionic product and lower dielectric constant, both of which enhance the release of organic compounds from biomass and increase the solubility of non-polar compounds [47].

Similar or better extraction yields and protein purities have been obtained through these extraction methods compared to alkaline and acid-based extractions (Table 3), indicating the suitability of these methods to act as chemical-free and environmentally friendly alternatives for protein extraction. For example, using hydrothermal extraction, Qin et al. [39] obtained an extraction yield of 66% and protein purity of 53% after treating BSG at 60 °C for 24 h. Applying subcritical conditions (185 °C/150 min), Alonso-Riaño et al. [47] solubilized a higher amount (~78%) of the proteins in BSG. Heating, however, either at high temperatures of 180–185 °C for 150 min or at low temperatures of 60 °C for longer time frames of up to 24 h, might denature the proteins, affecting their functionality. Furthermore, these parameters also raise concerns about the overall energy consumption and efficiency of the process. Recycling water, however, could potentially help to make the process more sustainable.

### 2.6. Enzymatic Extraction

Hydrolysis of BSG with enzymes such as carbohydrases and proteases aims to facilitate protein solubilization through the breakdown of carbohydrates and proteins resulting in the formation of protein hydrolysates [44,53,55]. A wide range of extraction yields ranging between ~31 and ~86%, have been reported in the literature, with the protein purity being ~40% in most extracts (Table 3).

The addition of carbohydrases and proteases can either be carried out as a one-stage process or separately as a two-stage process [48,53,55]. In a two-stage process, carbohydrases are typically first added to hydrolyze the cellulose and hemicellulose of BSG to facilitate the protein release. In the second stage, proteases partially break down the protein matrix, which increases protein solubility. However, no differences in protein recovery and yields were noticed between these approaches (Table 3). In general, the process of enzymatic extraction typically involves the formation of a 5 to 10% BSG dispersion that is incubated with 0.5 to 7.5% of carbohydrases, proteases, or both at optimum conditions. Adjustment of the mixture’s pH is often needed to achieve optimum enzyme activity, as the carbohydrases and proteases commonly require acidic and alkaline conditions, respectively. Some proteases, however, may require acidic or near-neutral conditions for optimum functionality. For example, Acid Protease A from *Aspergillus niger*, and Promod144 papain, from papaya fruit, work at pH 3.5 and 6.5, respectively. The mixtures are incubated at 50 to 60 °C for 3 to 5 h (depending on the enzyme used) and the solubilized proteins are recovered through centrifugation [53]. While some studies performed thermal inactivation of enzymes after completion of the incubation period [48,55], drying the final extract at mild or high temperature after protein recovery could also suffice to inactivate the enzymes added for the extraction [43,44,54].

The enzyme concentration, pH of the mixture, and incubation time were identified as the main factors critical to achieving high extraction yields. In general, an increase in the enzyme load led to higher extraction yields. Yu et al. [54], for instance, reported an increase in the extraction yield from ~35 to ~65% when the Alcalase concentration was increased from 1 to 40 μL per gram of dry BSG. Similar results were obtained in the study by He et al. [44], where an increase in the process yield from 77 to 84%, was reported upon increasing the protease concentration from 5 to 35 μL per gram of dry BSG. No significant differences were, however, identified between the purity of extracts. Moreover, 20 μL per gram of dry BSG was recommended as the optimal enzyme load in both these studies, considering there were no significant differences in the yield with the highest enzyme load evaluated.

The incubation time is another important parameter that has been reported to affect the extraction yield. In the presence of Alcalase at 10 μL per gram of dry BSG, Yu et al. [54] observed that the protein recovery increased from ~23% after 0.25 h to ~60% after 8 h. No significant differences were found in the extraction yields when the incubation time further increased to 24 h. Similarly, a non-linear trend in the enzymatic extraction yield was observed by Niemi et al. [53]. In this study, 66% of the protein was solubilized due to the protease activity after 30 min, whereas with longer incubation the solubilization effect of the protease decreased. After 7 h, only 50% of the protein solubilization could be related to the enzyme (i.e., 50% of the protein would also be solubilized without the protein). The study suggested that the release of carboxylic acid groups decreased the pH of the mixture over time, which resulted in conditions that were less favorable for the protease. The optimal time for extraction, therefore, depends on the enzyme class and also on the environmental condition of the mixtures during the extraction.

The main challenge in combined treatments of carbohydrases and proteases is the low purity of the extracts at ~40%. The reason for this is that carbohydrases solubilize small molecular weight sugars from BSG. Microfiltration of the liquid extracts after enzymatic treatments could separate sugars from the proteins and thereby increase their purity. For instance, Frederix and Greden [43] obtained extracts with 80–85% of protein purity after microfiltration with 0.5 to 2 kDa. Finally, considering that extraction of BSG proteins resulted in hydrolysates, studies of their functionality are critical to evaluate their applications in the food sector [42,48,55,62].

### 2.7. Cascade Extraction Methods

Low protein purity in the final extract with an average of ~50% remained a common challenge across various extraction methods studied. As discussed before, this is typically related to the hydrolysis and solubilization of carbohydrates. Adding processing operations such as centrifugation or membrane filtration to separate these compounds from the solubilized proteins seems to be a viable solution to increase protein purity. Additionally, the combination of different extraction methods could also help to achieve a synergistic effect on the yield and purity of proteins derived from BSG. Mixed results have, however, been reported, with a wide range of protein yields (43 to 86%) and purities (58–65%) being obtained through cascade extraction methods (Table 3).

Multi-step extraction approaches such as acid−alkaline and enzyme−alkaline techniques did not necessarily demonstrate a positive effect on extraction yield. For example, while an increase in protein recovery from ~50 to 65% was observed upon adding an acid pretreatment prior to alkaline extraction [46], cellulase pretreatment prior to alkaline extraction decreased protein recovery from 66% to 50% [40]. These results show that processing parameters have to be carefully selected to achieve and maintain optimum protein solubility.

Ultrasound technology has recently been studied as a method to enhance the solubilization of proteins prior to or during alkaline or enzymatic extraction by the formation and collapse of bubbles and sonolysis of water [41,42,46,54]. The use of ultrasound, in general, positively influenced the extraction yields. Li et al. [42] increased the protein extraction yield from 46 to 86% after adding ultrasound at 250 W for 20 min during alkaline extraction. When used prior to enzymatic extraction, ultrasound facilitated the extraction and enabled a reduction of 73% in enzyme use. Moreover, the processing time decreased by 56% [54]. Thus, there is considerable potential to combine physical and enzymatic methods to increase the efficiency of the process, but actual economic data are missing at the moment that would allow a more definite conclusion.

The use of microwaves, like ultrasound, during alkaline extraction also helps to enhance protein recovery. Aiming to induce intracellular heating and disruption of the cell walls of BSG, Barrios et al. [56] applied microwaves during hydrothermal extraction and achieved an extraction yield of ~90% using a maximum microwave power of 1800 W at 110 °C for 10 min. The study concluded potential feasibility for industrial scale-up after economic evaluation. In addition, the pulsed electric field technique also aims to disrupt the BSG matrix by electroporation of the cell membranes, hence increasing protein recovery [57]. However, Kumari et al. [57] did not observe any enhancement effect on the protein recovery and achieved low purity of the extracts due to the presence of large amounts of carbohydrates [57].

On a final note, while alkaline extraction is currently the most established method of protein extraction [44], chemical-free extraction methods such as hydrothermal and subcritical water extraction may pave the way toward more sustainable processing. The use of energy, water, and the emissions generated from the extraction processes, however, are also important considerations for sustainable upcycling of BSG. To date and to the best of our knowledge, no study has reported these factors during the evaluation of methods and approaches to extracting proteins from BSG. Optimization studies that were included in this review [38,39,42,45,47,49,53] could help to improve extraction efficiencies, minimize the use of resources, and lower the environmental impacts. Additionally, the advantages and disadvantages of different extraction methods, which have recently been reviewed by Rodriguez et al. [32], Piercy et al. [33], and Wen et al. [34], coupled with their ease of industrial use and scalability, could help to establish the most appropriate extraction method for BSG proteins.

## 3. Influence of Processing and Extraction Conditions on Protein Composition

The compositional and structural properties of the extracted BSG proteins vary with the raw materials used, the brewing process, and the extraction methods and conditions. A broad molecular weight distribution ranging from <5 kDa to >250 kDa has therefore been reported in the literature for BSG proteins, with most extracts being dominant in B, C, and ϒ hordeins (Table 4).

Although barley has traditionally been used to produce beer, a variety of starch-rich materials including other cereals, pseudo-cereals, or vegetables are currently being used in conjunction with barley. The type and content of proteins present in BSG, therefore, differ significantly. For instance, BSG obtained from cereals like barley and wheat is expected to be naturally rich in prolamins (43–80%), which is in contrast to oats and rice, where globulins (70–80%) and glutelins (64–74%) form the major storage proteins [16,63,64,65]. Further, the native prolamins of various cereals differ on a structural and molecular level [66], adding variability to the protein profile of the obtained BSG protein extracts.

The processes of malting, mashing, and kilning involved in the brewing process considerably influence the barley protein profile when it is transformed to BSG. Celus et al. [16] studied the impact of malting and mashing on the composition of the Osborne protein fractions obtained from barley and BSG. The authors reported that hordeins formed 43% of the total protein in both fractions. BSG, however, contained a higher amount of glutelin (22% vs. 14% in barley), and a lower number of albumins and globulins compared to barley (7% vs. 26% in barley). This was expected as the majority of water-soluble proteins go into the wort. Further, the protein type present in barley and BSG as hordein and glutelin differed. The BSG hordein extract contained only B and C hordeins, with the latter dominating the protein profile. In contrast, the barley hordein extract contained all hordein types, including A, B, C, D, and ϒ hordeins, with B hordein being the dominant protein. Similarly, the glutelin fraction from BSG only contained B hordeins compared to the barley glutelin fraction, which in addition also contained D hordeins. Similar results were reported through other extraction methods (Table 4), except Li et al. [42], who reported the presence of A-hordeins and suspected the presence of protein Z (an albumin), based on a band at 43 kDa, in a BSG extract obtained through ultrasound-assisted extraction. Further analysis through LC-MS is, however, needed to validate this hypothesis, as other proteins including B and gamma hordeins could also contribute to this molecular weight band owing to their similar migration profiles in SDS PAGE [67,68].

The extent of heating, either during kilning at temperatures of up to 200 °C after malting, or during wet protein extraction, is known to cause protein denaturation and aggregation. For instance, Kumari et al. [57] reported the presence of higher molecular weight proteins of 18 and 247 kDa in dark BSG, compared to that of 13 kDa in pale BSG, attributing it to protein aggregation. In contrast, a higher amount (~86–88%) of smaller proteins with MWs less than 5 kDa were found in black/dark BSG compared to pale/light BSG (~21–33%) by Connolly et al. [37]. This study also reported a 12% increase in these smaller molecular weight proteins extracted from pale BSG when the extraction temperature was increased to 50 °C from 20 °C. The heating temperature, therefore, needs to be carefully regulated, to minimize the protein denaturation desirable for optimum protein functionality.

The extraction techniques and their conditions further influence the composition and structure of the obtained protein fractions (Table 4). Ervin et al. [50], for instance, reported the presence of at least six different molecular weight fractions in salt extracts obtained at 100 °C, compared to at least 5 different fractions at 75 °C. Further, higher molecular weight fractions were observed at 100 °C compared to 75 °C, with the authors attributing this behavior to greater protein solubilization at a higher temperature. The higher molecular weight fractions, however, could also correspond to the protein aggregates resulting from protein denaturation at higher temperatures. Although alkaline extraction followed by isoelectric point precipitation has been the most common wet extraction technique used for protein isolation (Section 2), a few recent studies also combined ultrasound and/or enzymatic breakdown with alkaline extraction and acid precipitation to increase the protein yields [41,42]. These combinations, however, were found to alter the native protein structure. Li et al. [42] varied the ultrasound power (150–350 W), time (5–25 min), and duty cycle (20–100%), and observed that each of these variables modified the protein conformation to different degrees. While the duty cycle significantly affected the tertiary structure, the ultrasound power and time had an influence on the protein secondary structure. The primary structure of proteins remained unchanged, with thicker protein bands occurring after the ultrasound extraction, which is consistent with higher extraction yields (Section 2). On the contrary, the use of enzymes altered the molecular weight distribution of proteins. For instance, alkaline proteases hydrolyzed the native BSG proteins to molecular weights < 15 kDa, while proteins up to 43 kDa were obtained in the presence of acid proteases [53,54]. Ultra/nanofiltration has also been used in addition to proteolysis to obtain highly soluble protein fractions within a narrow MW range (2–4.5 kDa). This fraction was proposed to be suitable for beverage applications, which is in stark contrast to the predominantly water-insoluble prolamins present in BSG. The choice of the extraction method coupled with tailored conditions can therefore help to achieve the desired structure/molecular weight distributions of the extracted proteins to aid specific functionality.

## 4. Physicochemical Properties

Physicochemical properties such as solubility, denaturation temperature, charge, interfacial activity, and others are important characteristics of proteins to determine their functional behavior in food matrices. Some of these properties have been determined for proteins found in BSG and will be discussed below.

### 4.1. Protein Solubility

Protein solubility is the main physicochemical property that determines the suitability of proteins to be used in different food formulations at the required environmental conditions. Depending on the conditions (pH, ionic strength, temperature), proteins need to be selected that remain soluble or undergo a solubility transition. For example, liquid foods typically require a protein that remains soluble, whereas proteins often form a three-dimensional network by controlled desolubilization and aggregation in solid foods. Thus, it is important to understand protein solubility under different environmental conditions.

Several authors have investigated the solubility of the proteins found in BSG under different conditions. In general, BSG proteins have a very low solubility in the pH range that is relevant for food applications (pH 2 to 9) [36,37]. Celus et al. [36] reported a protein solubility below 8% over the pH range of 2 to 8.5 (Table 5). These findings were expected because of the protein composition of BSG (Section 2), which mainly consists of water-insoluble prolamins and glutelins [16,69]. This is because the soluble protein fraction solubilizes into the liquid phase during the brewing process, whereas the insoluble proteins end up in the BSG. The amount of solubilized proteins remains fairly constant after the protein rest and is only minimally affected by the subsequent thermal treatments during the wort preparation [70].

**Figure 2:**
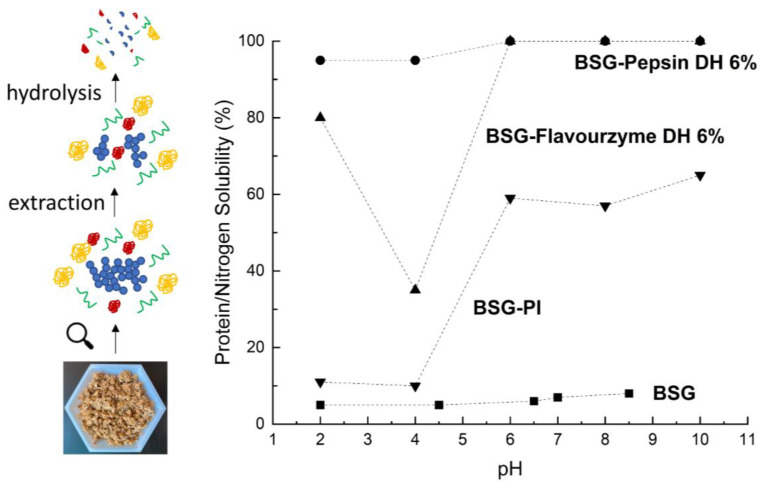
Proteins found in brewer’s spent grain have low solubility, but protein extraction and enzymatic hydrolysis considerably enhance protein solubility over a broad pH range. Data approximated from Conolly et al. [37], Bi et al. [80], and Marcus and Fox [81].

Because of the low solubility of BSG proteins, several authors studied the effect of protein extraction and hydrolysis on the solubility of proteins found in BSG [36,48,71] (Table 5). Here, the protein solubility of an extract obtained through the alkaline extraction−acid precipitation route is higher compared to untreated BSG. This is most likely because minor fractions of the water-soluble proteins found in BSG solubilize during the extraction process, and exhibit a different solubility profile than the major prolamin/glutelin fraction [71]. Moreover, it is known that hydrolysis occurs during the alkaline−acid precipitation route, which may have modified the solubility profile of the prolamin and glutelin fraction [79]. However, the solubility of alkaline extracted BSG proteins is still rather low (<70% in pH range 6–12) [71,72], and if high solubilities are needed, enzymatic hydrolysis proved to be an effective method to increase the solubility considerably [36,48,54,71] (Figure 2).

Celus et al. [36] established that especially pepsin is efficient in increasing the protein solubility of proteins that were present in a BSG extract and is superior to alcalase and flavourzyme at equivalent degrees of hydrolysis. Pepsin treatment at a 6% degree of hydrolysis resulted in a solubility of >90% in the pH range of 2–10 without a clear solubility minimum. This is in contrast to other enzyme treatments like Promod 144 MG, Alcalase 2.4 L, Corolase PP, Flavourzyme 500 L, Protamex, and fungi hydrolysis, which generally resulted in protein solubility curves with a minimum in the pH-range 3.5 to 4.5 [36,71,72]. However, the combination of exo- and endopeptidases has been shown to have a greater effect on the solubilization of protein compared to the use of individual enzymes, but it has to be considered that extensive hydrolysis results in smaller peptides and free amino acids that exhibit very different functionality (emulsification, foaming, gelation) than their high molecular counterparts [48,82].

### 4.2. Thermal Stability

The thermal stability of proteins is important during many food processing operations. The unfolding of proteins affects the ability of proteins to remain in solution or to form gels. Thus, it is important to understand at which temperature the proteins found in BSG denature. This is commonly measured using differential scanning calorimetry, but the available data on the thermal stability of BSG proteins are very limited. Most studies investigated glass melting/denaturation temperatures of the protein fractions that are also present in BSG in a dry state, such as hordein. One study investigated the denaturation temperatures of a barley protein extract that was mainly comprised the hordein and glutelin fraction (which is similar to BSG). The authors revealed a denaturation temperature *T*_d_ of 125 °C and a glass transition temperature *T*_g_ of 60.05 °C for a dry powder [83]. Other authors [84,85,86] have reported similar values, with one study even reporting a denaturation temperature of *T*_d_ = 163.5 °C [84]. It has to be mentioned, however, that all these data have been obtained for a dry powder only and denaturation temperatures are considerably influenced by the presence of water, with lower water activities typically resulting in an increase in denaturation temperature [87].

Interestingly, one study investigated the denaturation behavior of a protein isolate obtained from BSG at a concentration of 5% but no peaks were detected in the temperature range of 20–120 °C [78]. According to the authors, this was in line with other studies that reported no endothermic peak for prolamin and glutelin fractions. However, it remains unclear whether these effects might be influenced by the beer brewing and extraction process and more research is necessary to elucidate this phenomenon.

### 4.3. Surface Hydrophobicity

Surface hydrophobicity characterizes the exposed hydrophobic residues in a protein and is a function of the protein structure and amino acid composition. Limited studies have characterized this property as a function of extraction methods and conditions. A recent study found that the surface hydrophobicity of BSG proteins increased with increasing extraction temperature and pH during alkaline extraction, probably owing to protein unfolding and denaturation under these conditions [73]. Interestingly, the surface hydrophobicity of a commercial enzymatically hydrolyzed BSG protein isolate could not be quantified due to the breakdown of the majority of the native BSG proteins into smaller peptides of molecular weights < 14 kDa [74].

## 5. Techno-Functional Properties

Techno-functional properties that derive from the physicochemical properties are important to form different food structures, such as emulsions, foams, and gels. In this section, we will review the ability of proteins found in BSG to stabilize such food matrices.

### 5.1. Water and Oil Absorption Capacity (WAC/OAC)

Higher water and oil holding capacities are desirable in viscous and/or gelled food product applications like mayonnaise, yogurt, and plant-based meat applications. While water retention helps to reduce moisture loss during cooking, specifically in plant-based meat applications, the presence of fat enhances the mouthfeel and helps to retain the flavors in food products [73].

In general, BSG proteins exhibit better WACs (3–5 g/g) (Table 5) compared to other cereal and pulse proteins, including those from wheat, oat, pea, and soy (1.1–2.8 g/g) [73]. The extraction methods and conditions, however, are known to influence the WACs of the resulting isolates. Silva et al. [73], for instance, found that WACs of alkaline extracted BSG protein concentrates significantly increased when the pH was raised from pH 8 to 12 during the extraction process. The authors, however, attributed this increase to the associated water-insoluble dietary fibers that were present in these protein concentrates rather than the proteins, as the protein purities of these extracts varied between 28 and 44% *w*/*w*. Similarly, Li et al. [42], who used sonication prior to alkaline extraction, obtained significant variations in the WACs upon varying the ultrasonic power, sonication time, and the number of duty cycles. With an increase in the ultrasonic power (150 to 350 W) and sonication time (5 to 25 min), the WAC decreased and reached a minimum at 250 W and 15 min, after which it increased. While the initial decrease in WAC was associated with increasing hydrophobicity upon sonication, the following increase was thought to be a result of the hydration of partly denatured and dissociated proteins.

OACs ranging from 2 to 5 g/g have been reported in the literature for BSG protein extracts derived through different extraction methods (Table 5). Recent studies indicated that, although the extraction pH and temperature during alkaline extraction had no significant effect on OACs, the application of ultrasound prior to alkaline extraction positively influenced the OACs. Further, while the OAC of EverPro^®^ (EverGrain^®^ by AB InBev, St. Louis, MO, USA), a commercial BSG protein isolate produced via enzymatic hydrolysis, was lower compared to alkaline and ultrasound-assisted alkaline extracts (Table 5), this value was still higher than the OACs of commercial soy and pea protein isolates [74], indicating its suitability for use in fat-rich food matrices.

### 5.2. Emulsifying

The ability to stabilize the oil−water interface is of key importance for many food formulations [88]. BSG itself has not been utilized as an emulsifier yet but the derived extracts and hydrolysates have been tested for their emulsifying properties. The carried-out studies can be categorized into two categories. In the first category, the authors utilized a protein extraction method without further treatment for emulsion preparation, whereas in the second category, the authors treated the proteins further with hydrolyzing enzymes (Table 5).

Protein extracts from BSG obtained through ultrasound extraction and Osborne fractionation have been examined for their emulsion activity index (EAI) and emulsion stability index (ESI) by Li et al. [42] and Negi and Naik [75], respectively. Li et al. [42] observed that the EAI of a BSG extract obtained through ultrasound-assisted alkaline extraction resulted in an increase in EAI with increasing ultrasound power used during the extraction, whereas the ESI increased up to 250 W and then decreased again. The authors explained this phenomenon by previous observations that ultrasound treatments result in the unfolding of proteins and, as a result, in the exposure of hydrophobic groups that allow for faster adsorption onto the oil−water interface. However, it has to be mentioned that also the non-treated BSG extract showed some emulsifying properties in this study. In the second study by Negi and Naik [75], the authors fractionated BSG into its prolamin and non-prolamin fractions. Interestingly, the prolamin fraction showed inferior emulsion stabilization properties compared to the non-prolamin fraction, which might be due to the high hydrophobicity of the prolamins, which could result in a non-optimum balance of hydrophobic and hydrophilic surface areas [66]. Further investigations into the emulsifying properties of the non-prolamin fraction revealed rather large droplet sizes for emulsions prepared at pH 4, 7, 8, and 9 with droplet sizes of 6–7 μm, which was in contrast to bovine serum albumin at similar conditions which exhibited a maximum particle size of 2.5 μm at pH 4.

Thus, untreated BSG showed rather inferior emulsifying properties compared to other protein materials, and for this reason, other authors investigated the emulsifying properties of hydrolyzed BSG proteins (Table 5). In most studies, a protein extract was prepared using a single alkaline extraction or it was combined with ethanol or reducing agents to enhance the protein extraction yield [36,72,76,89]. These protein extracts were subsequently treated using different proteases. One study also treated the BSG before extraction with *Rhizopus oligosporus* [72]. However, in each case, the hydrolysis increased the EAI compared to the untreated protein extract [36,71,72,76,89].

For example, untreated BSG exhibited an overall low EAI, which was significantly increased by an alkaline extraction step and even further increased by hydrolysis. Especially a treatment with flavourzyme was able to increase the EAI from around 22 m^2^/g (BSG) to 78 m^2^/g (extracted BSG) to 110 m^2^/g (extracted BSG treated with flavourzyme to a degree of hydrolysis of 2%) [36]. Interestingly, the ESI remained rather constant in this study and could only be considerably enhanced by pepsin treatment when treated to a degree of hydrolysis of 6%. This is most likely related to the formation of different peptides that are being formed during hydrolysis that possess distinct surface-active properties and vary in their ability to stabilize interfaces depending on their surface chemistry and molecular weight. In general, there is an optimum molecular weight to achieve optimal emulsifying properties, and several authors reported lower EAIs [89] or ESIs [62] at increased degrees of hydrolysis, which was related to the formation of smaller molecular weight peptides. It was concluded that BSG hydrolysates should contain less than 40% of fragments with MW > 14,500 Da and should possess a high surface hydrophobicity S_0_ > 10 × 10^5^ [89].

In conclusion, the emulsifying properties of BSG are considerably changed upon hydrolysis and are influenced by the utilized enzyme. Depending on the obtained degree of hydrolysis, the formation and stability of the emulsion might be either increased or decreased. Especially limited hydrolysis with Flavourzyme and pepsin seem to be efficient in facilitating emulsion formation and stabilization, respectively.

### 5.3. Foaming

The ability of an ingredient to stabilize the air−liquid interface is another important functional property. Especially proteins are well known for their ability to form and stabilize foams [90] and therefore BSG has been investigated as a potential foaming agent. This is more challenging than one may expect and, similar to the previous discussion on emulsions, many authors utilized a protein extract or a hydrolysate to improve the foaming properties of BSG. In fact, early reports even suggested that a solvent extract of BSG can be used as an efficient *anti*-foaming agent with almost no foaming ability [36,91].

More recent studies that draw attention to the proteins in BSG reveal that the proteins extracted from BSG via the alkaline extraction route possess rather low foaming properties with especially low foam stabilization (<7 s) capabilities in the acidic pH range [77] but higher stabilities have been observed at pH 7 with around 30% of the foam still being present after 60 min for an alkaline protein extract [36] (Table 5). Higher stabilities have also been observed for a protein extract obtained through ethanol solubilization from barley itself. Yalçın et al. [92] reported foam half-times of their protein extracts of up to 450 s at pH 4, showing that the type of protein extraction highly influences the functionality. However, regarding BSG, several authors tried to improve foaming and foam stability through treatments such as ultrasound, hydrolysis, and electrostatic complexation with carrageenan:

- Li et al. [42] treated a BSG protein extract with ultrasound, which increased both foam formation (i.e., how much volume was generated) and foam stability. The results were attributed to changes in protein folding. Overtreatment decreased foam stability in this study.
- Proaño et al. [77] revealed that when a BSG protein extract is combined with λ-carrageenan the foam formation and foam stability are considerably increased. Foam expansion was almost doubled, and foam stability (half-time) was increased from a few seconds to 365 s for a formulation containing the protein extract and carrageenan at a 1:1 ratio at pH 3. The increased foam forming capabilities were related to particle formation due to associative complexation and an optimum balance of negative charges as well as hydrophobicity, which positively influenced film formation and thus foam stability.
- Celus et al. [36,89], Naibaho et al. [62], Connolly et al. [71], Vieira et al. [76], and Chin et al. [72] investigated how enzymatic/fungi hydrolysis affects the foaming ability of BSG proteins. Protein extraction generally enhanced the ability to form and stabilize foams, and hydrolysis further enhanced these capabilities in most studies (Table 5). Especially alcalase and pepsin have been shown to efficiently improve the foaming properties up to a degree of hydrolysis of 2–4% [36]. Higher degrees of hydrolysis typically decreased the foamability and foam stability [36,76].

In conclusion, foam formation and stability are considerably enhanced upon protein extraction from BSG and can be further tuned by hydrolysis, but the type of protease and degree of hydrolysis considerably affects the resulting functional property.

### 5.4. Solid Structure Formation

The ability to stabilize solid and semisolid food structures is an additional important functionality that determines the formation of structures such as gelled and extruded food products. Indeed, BSG and BSG protein extracts have been investigated as potential ingredients to structure solid food matrices. Most studies investigated the potential of using BSG in extruded food products, whereas a limited number of studies investigated how the proteins found in BSG could be used as a gelation agent.

**Figure 3:**
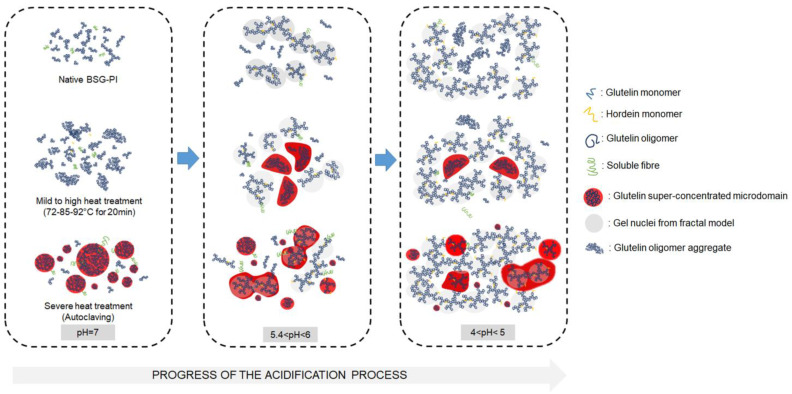
Semisolid gels can be formed from proteins found in brewers spent grains via an acid-induced gelation route. Heat-treatment of proteins before gelation results in glutelin-rich microdomains that alter the structural properties of the gel. Image adapted from Hellebois et al. [78] with permission of Elsevier. BSG-PI = Brewers spent grain protein isolate.

The comprehensive study of Hellebois et al. [78] revealed that a 5% protein concentrate (alkaline extracted) produced from BSG with a protein content of 75% was able to form a viscoelastic gel with glucono delta-lactone treatment that lowered the pH to 4.2. The elastic properties of the gel could be enhanced by adding a heat treatment (e.g., 72 °C/20 min) before the gelation step. This not only increased the storage modulus G’ but also the yield stress and flow point, whereas higher heat treatments were less effective in strengthening the gel. The authors also proposed which proteins are involved in the microstructure formation. The microstructure was mainly described as dominated by glutelin protein clusters with hordeins only playing a minor role in the structure formation (Figure 3). Upon controlled heating, small glutelin microdomains were observed that formed percolated gel structures and thereby enhanced the gel strength. Overall, this study demonstrated that BSG protein extracts are suited to form acid-induced gels similar to yogurt-style foods, but one limitation of this study is that protein extraction is necessary to form such structures. For this reason, other investigators tested how BSG behaves during extrusion processing.

BSG was utilized as an ingredient during extrusion processing to produce puffed snacks, pasta, breakfast cereals, different nutritional ingredients, and bread [8,93,94,95,96,97,98,99,100,101,102,103].

For expanded snack products, the addition of BSG causes a decrease in product expansion after the die, with a more dense macrostructure formed [95]. For example, the volume expansion index decreased from a maximum of 9.2 to 5.5 when 30% of BSG was added to milled rice flour [96]. Other authors observed similar effects [93,95,101]. This was explained by the fact that BSG contains high amounts of fibers that can influence the viscosity of the melt and absorb water, which decreases the amount of free water that is necessary for nucleation in the expansion process [96]. This lack of expansion might be somewhat counterbalanced by changing the processing parameters, such as increasing the temperature in the barrel. By doing so, expanded snack products enriched with BSG at concentrations of 10–30% can be produced to obtain snacks with more fiber and protein but the obtained color is typically darker with more brown tones [95,96].

BSG was also incorporated during cold extrusion processes, such as pasta production. When micronized (barley) BSG is added to semolina-based pasta only small amounts (5–10% depending on BSG origin) could be added until the sensory scores were negatively affected [97,104]. For this reason, other authors used approaches such as fermentation and air classification to treat the BSG before adding it to pasta [8,105,106]. Especially the addition of 15% of a protein-rich fraction from BSG that was obtained through air classification was rated as ‘excellent’—which was similar to the control—in a sensory panel. Moreover, the addition allowed for having enough protein and fiber to carry the claim of ‘High Protein’ and ‘High Fibre’ [8]. It could also be shown that by processing BSG using enzymatic digestion (carbohydrase) and a fermentation step with *Lactiplantibacillus plantarum* PU1 the sensory profile of BSG enriched (15%) pasta could be considerably enhanced. However, not all oral processing parameters—such as chewiness—could be mitigated and especially color was significantly altered towards more brown in all formulations [106]. Consequently, low amounts of raw BSG may be added to pasta formulations but BSG fractionation seems to be a promising technique to increase the amount that can be added to pasta.

Similar to pasta, a recent study compared the suitability of two different fractions obtained from BSG for bread making, namely a protein-rich and a fiber-rich fraction [107]. The researchers replaced up to 11% and 16% of regular flour with their fiber and protein rich BSG flour to obtain a bread that qualifies to be labeled as ‘High in Fiber’. The study showed that especially the fiber-rich fraction (which is mainly insoluble fiber, 63.8%) resulted in high-quality bread characteristics when flour was replaced at a level of 11%. For example, the specific volume was only slightly below that of the control (3.72 vs. 4.46 mL/g), the crumb texture was soft (9.03 N hardness), and the overall crumb structure was comparable to the control without BSG. The protein-rich fraction in contrast performed less favorably at higher concentrations, which was related to the different behavior of the proteins present in BSG compared to wheat-gluten proteins during baking, resulting in a considerable decrease in specific volume. Studies with whole BSG found similar results. When 10% BSG was added to wheat flour, the specific volume was only decreased by a small margin, from 2.86 mL/g for the regular wheat bread to 2.69 and 2.50 mL/g when 10% BSG and fermented BSG were added, respectively [108]. The same study reported that a 10% addition of BSG to the dough was completely accepted by the sensory panel and that BSG is a promising ingredient to increase the protein, dietary fiber, and mineral levels in bread. This reveals that highly functional fractions can be obtained from BSG that need to be tailored to specific food applications.

Lastly, BSG was added to other solid foods to partly replace other ingredients to increase the fiber and protein content as well as to fortify the products with compounds that possess antioxidative properties such as ferulic and phenolic acids [109,110]. Indeed, studies made with cookies showed that BSG can be incorporated into such food matrices. The addition of BSG resulted in cookies that exhibited an increased hardness but up to 20% BSG could be successfully incorporated without significantly altering the overall sensory acceptability [110]. Moreover, the glycemic index decreased from 81.1 to 74.7 with the addition of 20% BSG, showing that there are some potential nutritional benefits for BSG-enriched foods.

Overall, fortification of different solid foods with BSG seems to be a promising tool to add more fiber and protein to different formulations, but the sensorial and functional properties are typically modified upon addition, which often limits the amount that can be added to the food.

## 6. Nutritional Properties: Amino Acid Profile and Protein Digestibility

The amino acid profile along with the protein digestibility act as the two key determinants of the nutritive value of a protein. Glutamine and proline form the most abundant amino acids in BSG protein extracts, constituting 30–38.5% (*w*/*w*) of the total amino acids. This is in line with these being the main amino acids of barley hordeins [111]. Further, these extracts contain all essential amino acids, with leucine, phenylalanine, and valine being the most abundant amino acids, each comprising around 4 to 7% (*w*/*w*) of the total protein [37,44,45]. While lower quantities of 0.4–1.5% (*w*/*w*) have been reported in BSG protein extracts for each of the S-containing amino acids methionine and cysteine [37,44], a recent study by Jaegar et al. [74] indicated that their cumulative content (i.e., methionine + lysine) was higher than what is typically found in pea and soy protein isolates and was sufficient to fulfill the recommended essential amino acid requirement per gram of protein outlined by the World Health Organization. Lysine, an essential amino acid commonly deficient in the majority of cereals, has been found to occur at a concentration of ~3%, meeting 80% of the daily essential amino acid requirement [37,44,74]. These results suggest that combining BSG proteins with other lysine-rich proteins, such as those from soy and pea [74], along with obtaining high lysine cereal varieties through plant breeding [112], may aid in achieving a more balanced and nutritive amino acid profile of these extracts. It is important to note that all the aforementioned amino acid contents are for pale BSG. Roasting of barley grains at high temperatures of up to 200 °C during the kilning process to obtain roasted BSG (see Figure 1) considerably reduces the total and free amino acid content of protein extracts, owing to the participation of proteins in Maillard reactions at such high temperatures [37,57].

Further, the extraction methods and conditions are known to influence the type and content of amino acids present in the BSG protein extracts:

- Temperature has been shown to influence the protein yield for different extraction methods. Connolly et al. [37], studied the impact of extraction temperature during alkaline extraction of BSG proteins and obtained a higher total amino acid content at 50 °C compared to 20 °C. Similarly, the total amount of free amino acids during subcritical water extraction was found to increase with increasing temperatures (125 to 160 °C) and extraction times (0 to 240 min) [47]. Additionally, amino acid profiles differed with extraction temperatures during salt extraction. Ervin et al. [50], for instance, found higher levels of glutamic acid and proline, but lower levels of the other amino acids in extracts produced at 75 °C compared to 100 °C.
- He et al. [44] studied the differences in the amino acid profiles of extracts obtained through alkaline, reducing agent, and enzymatic extraction approaches, and found higher amounts of lysine (4.1%, *w*/*w*) when an alcalase mediated extraction was used compared to alkaline extraction (3.1%, *w*/*w*). Further, the type of enzyme used during extraction also impacts the overall amino acid profile. Krissa et al. [48], for instance, showed that Flavourzyme (exopeptidase) and Protamex (endopeptidase) acted synergistically to enhance the availability of hydrophobic amino acids, which initially was much lower when each of these enzymes was used alone.
- The impact of different pretreatments was reported by Qin et al. [39], who showed a 33% increase in the total amount of amino acids when a diluted acid pretreatment using sulfuric acid was utilized to extract proteins instead of a chemical-free hydrothermal approach.
- While the use of different deep eutectic solvents (DES) did not alter the overall amino acid profile, lower lysine contents in the protein extracts and residues were obtained after DES treatment compared to raw BSG. This could potentially be related to lysine degradation or fragmentation into smaller molecular weight peptides that could not be retained during the dialysis step [45].

In terms of digestibility, barley protein has the highest protein digestibility corrected amino acid score (PDCAAS) value of 0.55, compared to other commonly used grains in beer production, including wheat, corn, and rice, having values of 0.41, 0.43, and 0.47 respectively [113]. The PDCAAS of BSG and the derived protein extracts have not been characterized yet in the literature. BSG, however, may have a higher protein digestibility than barley as the proteolytic activity during malting and mashing increase the content of free amino acids, which are known to absorb more readily compared to intact proteins [5,114]. Compared to barley, however, some anti-nutritional factors are enriched in BSG such as polyphenols. BSG was reported to contain 131 mg/L of polyphenols compared to 90 mg/L in barley [108]. These polyphenols may bind to proteins and reduce their digestibility [115]. Extraction of proteins, therefore, seems to be a viable strategy to reduce the content of polyphenols, but actual data are missing at the moment. Further, enzymatic breakdown during the extraction process, which is often done to obtain soluble protein isolates, has been shown to enhance the availability of hydrophobic amino acids [48], suggesting higher digestibility. Future studies in this direction are, however, needed to estimate and understand the factors affecting the digestibility of protein extracts produced from BSG.

## 7. Current Market Applications

BSG has been extensively studied and several potential applications have been proposed over the years, which have been recently reviewed by Chetrariu and Dabija [116]. However, the products that have been released onto the market that contain upcycled BSG are still rather limited. The company Evergrain (Wilmington, DE, USA) released different products based on BSG that have been obtained from barley−rice mixtures. Their current product EverPro^®^ has around 85% protein and is most likely obtained by a combination of enzymatic hydrolysis to break down the starch and proteins followed by centrifugal separation and micro- as well as nanofiltration steps to purify the resulting proteins [43]. According to their patent, the molecular weight distribution of the proteins is between 300 Da and 30 kDa. The company mainly advertises this product for beverage applications given its high solubility.

Other companies such as Regrained^®^ (San Francisco, CA, USA) sell BSG-enriched flour mixes for brownies, pizza, bread, and cakes. In addition, their portfolio contains pasta, puffs, and bars containing their BSG product. The company patented a BSG processing operation based on intermittent infrared drying combined with stirring that yields a final product with a lower microbial load, increased crispiness, and a more pleasant flavor [117].

While the current industrial applications of BSG protein isolate are limited, its appreciable water and oil holding capacity (Section 5.1), coupled with the gel-forming ability (Section 5.4), indicate its potential to be used in plant-based meat, gelled dairy alternatives (e.g., yogurt) and desserts, and fat-rich dressings.

## 8. Conclusions

The large amount of BSG produced makes it a promising material for food applications. Research efforts over the last years has shed more light on the optimization of extraction methods, the protein composition, and the functionality of the proteins found in BSG. Enzymatic treatments and fractionation methods seem to be promising techniques to obtain functional protein extracts that can be coupled with alkaline extraction methods. Solubility, emulsifying, and foaming properties can be increased by hydrolysis approaches, whereas untreated or fractionated BSG can be used in solid foods such as pasta, bread, and cookies. A major drawback of the current state of knowledge is that many studies do not report the raw material composition of BSG, which limits some fundamental understanding of its properties. Future research towards the incorporation of these proteins in high-protein plant-based products and their bioavailability in these matrices would further aid in expanding their utilization on a commercial level.

## Figures and Tables

**Table 1:** Composition of brewer’s spent grain [4,5,6,7].

Protein	Fiber	Lipids	Starch	Ash
15–30%	50–80%	3–10%	1–12%	2–5%

**Table 2:** Composition and structure of barley proteins.

Barley Proteins	Content	Mono/Di/Polymer	MW Range (kDa)	Theoretical MW (Da) **	Number of Amino Acids **	Negatively: Positively Charged Residues **	Amino Acids (%) **		Theoretical pI **	GRAVY **	Aliphatic Index **	UniProtKB Reference *
							Cys + Met	Lys				
Prolamins and Glutelins *	35–55% and 35–45% [11]		10 → >100									
D-hordein	2–4% [17]	Polymers or aggregated with B-hordeins [18,19]	>100 [18]	80,410	757	19/21	1.8 S-poor [12,13]	1.2	8.10	−1.104	37.70	Q84LE9
C-hordein	10–20% [18]	Monomers [18,19]	55–75 [18]	36,508	310	4/4	1.3 S-poor [12,13]	0.3	6.81	−1.225	40.55	Q41210
B-hordein	70–80% [18]	Monomers or polymers or aggregated with D hordeins [15,18]	35–50 [18]				S-rich [12,13]	n.d.				
B1				33,422	293	5/10	4.1	1.0	8.86	−0.554	74.16	P06470
B2				n.d.	n.d.	n.d.	n.d.	n.d.	n.d.	n.d.	n.d.	n.d.
B3				32,412	284	8/9	4.6	0.7	8.16	−0.533	79.58	I6SW30
γ-hordein	1–2% [17]	Monomers or polymers [18]	35–45 [18]				S-rich [12,13]					
γ1				34,737	305	8/11	5.6	2.0	8.34	−0.497	71.81	P17990
γ2				n.d.	n.d.	n.d.			n.d.	n.d.	n.d.	
γ3				33,189	289	10/9	5.2	1.7	6.70	−0.762	68.10	P80198
A-hordeins/Low Molecular Weight Hordeins	n.d.	n.d.	10–25 [14,15]	n.d.	n.d.	n.d.	S-rich [14]		n.d.	n.d.	n.d.	n.d.
Albumin/Leucosin	11% [20]		<14 to 58 [21]									
α-Amylase	n.d.	n.d.	45 [22]	47,403	427	53/43	2.3	5.9	5.92	−0.312	83.19	Q40015
Protein Z		Dimer and tetramer ^10^	40 [20,23]									
Z4	5–7% [20]			43,276	399	42/34	2.3	5.5	5.72	0.040	96.09	P06293
Z7				42,821	397	42/33	2.1	5.0	5.45	0.071	96.07	Q43492
ZX				42,947	398	42/41	2.0	7.3	6.77	−0.014	89.45	Q40066
Lipid transfer proteins	∼5% [24]	n.d.	7 or 9 [20]	9694.96	91	6/8	9.9	4.4	8.19	−0.385	74.95	O81135
Globulin or Edestin (α, β, ϒ, and δ)	15% [20]	n.d.	<14 to 53 [21]	24,560	224	21/28	6.3	2.2	9.04	61.79	−0.664	Q84NG7

* Glutelin fraction majorly contains only B and D hordeins [16]. n.d., no data. GRAVY: Grand average hydropathicity. Met: Methionine; Cys: Cysteine; Lys: Lysine. Obtained from UniProt (https://www.uniprot.org/uniprotkb accessed on 20 December 2022); ** Calculated using ProtParam (https://web.expasy.org/protparam accessed on 20 December 2022).

**Table 3:** Methods and conditions to extract proteins from BSG.

Type of Extraction	Pretreatment of BSG	Main Extraction Parameters	Extraction Yield *	Protein Purity	Reference
Alkaline	4.8% *w*/*v* Pale BSG−water; shearing 24,000 rpm, 2 min; incubation 1 h, room T, stirring	0.11 M NaOH; incubation 50 °C, 1 h; centrifugation 2700× *g*, 10 °C, 20 min; liquid recovery; acid precipitation 2 N HCl, pH 3.8; incubation room T (n.d.), 15 min; centrifugation (n.d.); pellet recovery; addition water, 2 N NaOH, pH 7; freeze drying	59%	46%	[37]
	12.5% *w*/*v* BSG−water; shearing (n.d.), 1 min	1 M NaOH, pH 11–12; incubation T (n.d.), 60 min; centrifugation 3800× *g*, 4 °C, 15 min; liquid recovery; acid precipitation (n.d.), pH ~2.5; centrifugation (n.d.); pellet recovery; freeze drying	66%	n.d.	[40]
	n.a.	17% *w*/*v* BSG−water, 0.1 M NaOH; incubation 60 °C, 1 h; filtration 180 μm; liquid recovery; acid precipitation, 2 M citric acid, pH 4; centrifugation 10,000× *g*, 4 °C, 10 min; pellet recovery; freeze drying	41%	60%; dry basis	[36]
	Drying 60 °C, 6.5 h	(n.d.) BSG−solution 0.1 M NaOH; incubation room T (n.d.), 2 h; acid precipitation 2 M HCl; incubation refrigeration T (n.d.), 18 h; centrifugation 2700× *g*, 10 min; pellet recovery; drying 60 °C, until constant weight	~50%	69%	[46]
	5% *w*/*w* BSG-water; wet milling (n.d.), ×2, mean particle size 162 μm	5% *w*/*w* NaOH−BSG solution, (n.d.) M NaOH; incubation 60 °C, 4 h; sieve shaking-water filtration, 278 oscillation/min, 15 min, 75 μm; liquid recovery; drying 60 °C, 24 h	82% **	37%	[44]
	Drying 60 °C, until 5% moisture content; defatting 24% *w*/*v* BSG−hexane, 200 rpm, 37 °C, 1 h; centrifugation 4500× *g*, 25 °C, 10 min, repeat until colorless hexane; filtration Whatman grade 1; drying T (n.d.), until constant weight	5% *w*/*v* BSG−water, 0.11 M NaOH; incubation 50 °C, 1 h, 200 rpm; centrifugation 5000 rpm, 10 min; liquid recovery; repeat the extraction, ×2	78%	42%	[39]
	Washing with water, until neutral pH; drying 45 °C, 3 h or until 8% *w*/*w* moisture content	5% *w*/*v* BSG−water, 0.1 M NaOH; incubation with stirring (n.d.), 50 °C, 4 h; separation (n.d.); liquid recovery	51%	n.d.	[47]
	Drying 70 °C; milling (n.d.); sieving 385 μm	10% BSG−water, 2 M NaOH, pH 8.5; incubation 50 °C, 3 h, 150 rpm; centrifugation 4000× *g*, 15 min, liquid recovery	~18%	n.d.	[48]
	Autoclaving 121 °C, 15 min; drying 60 °C, until < 10% moisture content; micronization (n.d.), 125–250 μm	6.7% *w*/*v* BSG−solution 0.01 M NaOH, pH 12.4; incubation 60 °C, 30 min; centrifugation 3871× *g*, 25 °C, 15 min; liquid recovery; repeat the extraction, ×3; freeze drying	~45%	n.d.	[38]
	Autoclaving 121 °C, 15 min; drying 60 °C, until < 10% moisture content; micronization (n.d.), 125–250 μm; defatting 6.25% BSG−solvent mixture 50% *v*/*v* methanol−chloroform, 1 h, room T; vacuum filtration (n.d.); drying 60 °C, time (n.d.); delignification 20% BSG−solution 60% ethanol, 180 °C, 90 min; vacuum filtration (n.d.); solid recovery; washing with ethanol, ×3; drying 60 °C, time (n.d.)	6.7% *w*/*v* BSG−solution 0.01 M NaOH, pH 12.4; incubation 60 °C, 30 min; centrifugation 3871× *g*, 25 °C, 15 min; supernatant recovery; repeat the extraction, ×3; freeze drying	~38%	n.d.	[38]
Acids	n.a.	0.35% *w*/*w* BSG−acid solution, 4% *w*/*w* sulfuric acid; autoclaving 121 °C, 60 min; filtration (n.d.); liquid recovery	90%	24%	[39]
	Washing with water, until neutral pH; drying 50 °C, time (n.d.)	12.5% *w*/*w* BSG−acid solution, 1% (n.d.) sulfuric acid; autoclaving 130 °C, 26 min; liquid recovery	~63%	~24%	[49]
Reducing agent	5% *w*/*w* BSG−water; wet milling (n.d.), ×2, particle size (mean) 162 μm	5% *w*/*w* sodium bisulfite−BSG in solution, (n.d.) M sodium bisulfite; incubation pH 5, 60 °C, 4 h; sieve shaking-water filtration, 278 oscillation/min, 15 min, 75 μm; liquid recovery; drying 60 °C, 24 h	68%	39%	[44]
Salt	Drying (n.d.); milling (n.d.), particle size 1 mm	5% *w*/*v* BSG−salt solution (3% sodium dodecyl sulphate + 0.5% Na_2_HPO_4_), pH 7, l h, 100 °C; filtration, liquid recovery; ethanol precipitation, 95% ethanol (0.7 mL per mL of extract), refrigeration 4 °C, 16 h; centrifugation 9500× *g*, 0 °C; ethanol wash; freeze drying.	49%	61%	[50]
	Drying (n.d.); milling (n.d.), particle size 1–2 mm	2.5% *w*/*v* BSG−salt solution (0.5% sodium dodecyl sulphate + 0.6% Na_2_HPO_4_), 91 °C, 98 min	~58%	n.d.	[51]
Deep eutectic solvents	Two-stage drying 50 °C, 1 h, 40 °C, overnight	10% *w*/*w* BSG−solvent, molar ratio 1:2 sodium acetate and urea; incubation 80 °C, 4 h; filtration 150 μm; liquid recovery; washing solids with hot water, T (n.d.), ×8; liquid recovery; blend of the liquids; membrane filtration (n.d.); liquid recovery; freeze drying	79%	54%	[45]
Pressurized solvent extraction	n.a.	(n.d.) BSG−solution 4.7% ethanol; heating 155 °C, 10 min, ×5; liquid recovery; centrifugation (n.d.); liquid recovery	69%	20%	[41]
Hydrothermal	Drying 60 °C, until 5% moisture content; defatting 24% *w*/*v* BSG−hexane, 200 rpm, 37 °C, 1 h; centrifugation 4500× *g*, 25 °C, 10 min; repeat until colorless hexane; filtration Whatman grade 1; solid recovery; drying T (n.d.), until constant weight	4% *w*/*v* BSG−water; heating 60 °C, 24 h, 250 rpm; cooling T (n.d.), until room T (n.d.); membrane filtration (n.d.)	66%	53%	[39]
	Drying room T, 3 days	25% *w*/*w* BSG−water; heating 180 °C, 40 min, filtration 250 μm	49%	n.d.	[52]
Subcritical water	Washing with water, until neutral pH; drying 45 °C, 3 h or until 8% *w*/*w* moisture content	12 g BSG, water 4 mL/min; autoclaving 185 °C, 5 MPa, static holding time 30 min, extraction time 150 min; extract recovery	78%	n.d.	[47]
Enzymatic	Drying T, time (n.d.); milling (n.d.); sieving 300 μm	10% *w*/*v* BSG−water, 100 U/g Depol 740 L; incubation 50 °C, 5 h; centrifugation 4000 rpm, 30 min; liquid recovery	~63%	n.d.	[53]
	Drying T, time (n.d.); milling (n.d.); sieving 300 μm	10% *w*/*v* BSG−water, 100 U/g Depol 740 L; incubation 50 °C, 5 h; centrifugation 4000 rpm, 30 min; pellet recovery; second hydrolysis 10% *w*/*v* solids, 10 U/g Alcalase 2.4 L, 0.05 M sodium carbonate and NaOH, pH 9.5; incubation 40 °C, 4 h; centrifugation 4000 rpm, 4 °C, 30 min; liquid recovery	~86%	n.d.	[53]
	Drying T, time (n.d.); milling (n.d.); sieving 300 μm	10% *w*/*v* BSG−water, 100 U/g Depol 740 L; incubation 50 °C; 5 h; centrifugation 4000 rpm, 30 min; pellet recovery; second hydrolysis, 10% *w*/*v* solids, 10 U/g Acid Protease A, 0.05 M sodium citrate, pH 3.5; incubation 40 °C, 4 h; centrifugation 4000 rpm, 4 °C, 30 min; supernatant recovery	~40%	n.d.	[53]
	Drying T, time (n.d.); milling (n.d.); sieving 300 μm	10% *w*/*v* BSG−water, 100 U/g Depol 740 L; incubation 50 °C, 5 h; centrifugation 4000 rpm, 30 min; pellet recovery; second hydrolysis 10% *w*/*v* solids, 10 U/g Promod 144 GL, 0.05 M McIlvaine’s buffer, pH 6.5; incubation 40 °C, 4 h; centrifugation 4000 rpm, 4 °C, 30 min; supernatant recovery	~31%	n.d.	[53]
	5% *w*/*w* BSG−water; wet milling (n.d.), ×2, particle size (mean) 162 μm	3.5% *v*/*w* Alcalase−BSG; incubation 60 °C, 4 h; sieve-shaking and water filtration 15 min, 75 μm, 278 oscillation/min, 120 mL water; liquid recovery; drying 60 °C, 24 h	83% **	43%	[44]
	Drying 60 °C. time (n.d.); milling (n.d.); sieving 840 μm	5% *w*/*v* BSG−water, 4% *v*/*w* Alcalase 2.4 L-BSG, 5 M NaOH, pH 8; incubation 110 rpm, 60 °C, 4 h; centrifugation 13,800× *g*, 4 °C, 10 min; liquid recovery; washing pellet with water, ×2; liquid recovery; blend of the liquids; drying 60 °C, time (n.d.)	~65% **	n.d.	[54]
	Drying 70 °C, time (n.d.); milling (n.d.); sieving 385 μm	9% *w*/*v* BSG−water, 0.5% Protamex-BSG, 2 M NaOH, pH 8.5; incubation 150 rpm, 50 °C, 3 h; inactivation 95 °C, 10 min; centrifugation 4000× *g*, 15 min; supernatant recovery	~58%	n.d.	[48]
	Drying 70 °C, time (n.d.); milling (n.d.); sieving 385 μm	9% *w*/*v* BSG−water; 0.5% Protamex−BSG, 0.1% *w*/*w* Flavourzyme−BSG, 2 M NaOH, pH 8.5; incubation 150 rpm, 50 °C, 3 h; inactivation 95 °C, 10 min; centrifugation 4000× *g*, 15 min; liquid recovery	~64%	n.d.	[48]
	10% *w*/*v* BSG−water; shearing 1 min, 24,000 rpm	7.5% *v*/*w* carbohydrases−BSG, pH 5; incubation 50 °C, 4 h; inactivation 80 °C, 20 min; centrifugation 2700× *g*, 10 °C, 10 min; liquid recovery; second hydrolysis of the pellet, 10% solution in water, Alcalase 2.4 L (2% *w*/*w* protein), Flavourzyme 500 L (1% *w*/*w* protein); incubation 50 °C, 4 h; inactivation 80 °C, 20 min; centrifugation 2700× *g*, 10 °C, 10 min; liquid recovery; 7% pellet-water; incubation 50 °C, 30 min; centrifugation 2700× *g*, 10 °C, 10 min; liquid recovery; blend of the liquids; freeze drying	63%	44%	[55]
	10% *w*/*v* BSG−water; shearing 1 min, 24,000 rpm	7.5% *v*/*w* carbohydrases−BSG, pH 5; incubation 50 °C, 4 h; inactivation 80 °C, 20 min; centrifugation 2700× *g*, 10 °C, 10 min; liquid recovery; second hydrolysis of the pellet, 10% solution in water, Alcalase 2.4 L (2% *w*/*w* protein), Protease P (1% *w*/*w* protein); incubation 50 °C, 4 h; inactivation 80 °C, 20 min; centrifugation 2700× *g*, 10 °C, 10 min; liquid recovery; 7% pellet-water; incubation 50 °C, 30 min; centrifugation 2700× *g*, 10 °C, 10 min; liquid recovery; blend of the liquids; freeze drying	53%	39%	[55]
	10% *w*/*v* BSG−water; shearing 1 min, 24,000 rpm	7.5% *v*/*w* carbohydrases−BSG, pH 5; incubation 50 °C, 4 h; inactivation 80 °C, 20 min; centrifugation 2700× *g*, 10 °C, 10 min; liquid recovery; second hydrolysis of the pellet, 10% solution in water, Prolyve 1000 (2% *w*/*w* protein), Protease P (1% *w*/*w* protein); incubation 50 °C, 4 h; inactivation 80 °C, 20 min; centrifugation 2700× *g*, 10 °C, 10 min; liquid recovery; 7% pellet-water; incubation 50 °C, 30 min; centrifugation 2700× *g*, 10 °C, 10 min; liquid recovery; blend of the liquids; freeze drying	59%	44%	[55]
	10% *w*/*v* BSG−water; shearing 1 min, 24,000 rpm	7.5% *v*/*w* carbohydrases−BSG, pH 5; incubation 50 °C, 4 h; inactivation 80 °C, 20 min; centrifugation 2700× *g*, 10 °C, 10 min; liquid recovery; second hydrolysis of the pellet, 10% solution in water, Corolase PP (2% *w*/*w* protein) and Flavourzyme 500 L (1% *w*/*w* protein); 10% solution in water; incubation 50 °C, 4 h; inactivation 80 °C, 20 min; centrifugation 2700× *g*, 10 °C, 10 min; liquid recovery; 7% pellet-water; incubation 50 °C, 30 min; centrifugation 2700× *g*, 10 °C, 10 min; liquid recovery; blend of the liquids; freeze drying	52%	43%	[55]
	Size reduction (n.d.); ~10% BSG−water	(n.d.) % Glucoamylase−BSG; incubation 50–65 °C, 15–60 min; second hydrolysis (n.d.) % alkaline protease-BSG, NaOH or KOH, pH 7–10; incubation 50–75 °C, 15–60 min or until ~10 degree of hydrolysis; inactivation 75–90 °C, 10–25 min; decantation-centrifugation (n.d.); liquid recovery; washing of solids (n.d.); blend of the liquids; microfiltration 0.03–0.5 μm; permeate recovery; nanofiltration 1–8 bar, 0.5–2 kDa; retentate recovery; vacuum evaporation (n.d.), to 55% solids; spray drying (n.d.)	n.d.	80–85%	[43]
Acid and alkaline	Drying 60 °C, 6.5 h; (n.d.) % BSG−water, 0.5% *w*/*v* sulfuric acid; autoclaving 100 °C, 20 min, 103.4 bar; extract recovery; 1% NaOH, pH 3, overnight; centrifugation 2700× *g*, 10 min; pellet recovery; drying 60 °C, 18 h	(n.d.) BSG−solution 0.1 M NaOH; incubation 40 °C, 60 min; acid precipitation 2 M HCl; incubation cooling T (n.d.), 18 h; centrifugation 2700× *g*, 10 min; pellet recovery; drying 60 °C, until constant weight	65%	60%	[46]
Enzymatic and alkaline	20% *w*/*v* BSG−water; shearing (n.d.), 1 min; 1.96% cellulase−BSG solution, 1 M HCl, pH 4.5; incubation 50 °C, 60 min; inactivation 85 °C, 20 min; centrifugation 3800× *g*, 4 °C, 15 min; pellet recovery	11% *w*/*v* BSG−water, 1 M NaOH, pH ~11.5; incubation T (n.d.), 60 min; centrifugation 3800× *g*, 4 °C, 15 min; liquid recovery; acid precipitation pH ~2.5; centrifugation (n.d.); pellet recovery; freeze drying	50%	n.d.	[40]
Ultrasound and alkaline	Drying 50 °C, time (n.d.); milling (n.d.); sieving 355 μm	6.7% *w*/*v* solid-solution 0.11 M NaOH; incubation with ultrasound 20–25 kHz, 250 W, 25 °C, 20 min, 60% duty cycle, pulses mode; centrifugation 8000× *g*, 10 °C, 20 min; liquid recovery acid precipitation 2 M HCl, pH 3.8; centrifugation 8000× *g*, 10 °C, 20 min; pellet recovery; solubilization 2 M NaOH, pH 7; dialysis 1000 Da, 4 °C, overnight (n.d.); freeze drying	86%	58%	[42]
	n.a.	5% *w*/*v* BSG−solution 0.11 M NaOH; incubation with ultrasound 70% amplitude 60 °C, 15 min, ×2; centrifugation (n.d.); liquid recovery; acid precipitation (n.d.) M HCl, pH 3.8; centrifugation 4000× *g*, 10 min; pellet recovery; freeze drying	43%	n.d.	[41]
	Drying 60 °C, 6.5 h; 5% *w*/*v* BSG−water; ultrasound 37 kHz, pulses mode, 30 °C, 20 min; decantation (n.d.); solid recovery	(n.d.) BSG−solution 0.1 M NaOH; incubation 40 °C, 60 min; acid precipitation 2 M HCl; incubation cooling T (n.d.), 18 h; centrifugation 2700× *g*, 10 min; pellet recovery; drying 60 °C, until constant weight	55%	~65%	[46]
Ultrasound and enzymatic	Drying 60 °C, time (n.d.); milling (n.d.); sieving 840 μm; 5% *w*/*v* BSG−water; ultrasound 227.5 W/L, pulse 5 s, 10 min	5% *w*/*v* BSG−solution 4% Alcalase 2.4 L, 5 M NaOH, pH 8; incubation 110 rpm, 60 °C, 4 h; centrifugation 13,800× *g*, 4 °C, 10 min; liquid recovery; washing pellet with water, ×2; liquid recovery; blend of the liquids; drying 60 °C, time (n.d.)	~70% **	n.d.	[54]
Microwave and alkaline	Drying 60 °C, until 3% moisture; milling (n.d.), particle size 1 mm; 10% *w*/*w* BSG−water	0.05 M NaOH; microwave 110 °C, 10 min, power 1800 W; centrifugation 10,000 rpm, 10 min; liquid recovery	92%	n.d.	[56]
Pulsed electric field and hydrothermal	16.7% *w*/*w* BSG−water; pulsed electric field application at 2.8 kV/cm, 3000 pulses, 20 μs pulse width	Aqueous extraction, 55 °C, 220 rpm, 16 h	n.d.	20–24%; dry basis	[57]

n.a., not applicable. n.d., no data. *, % of proteins extracted from total protein content in BSG. **, % of the protein in the supernatant divided by protein in the solids and protein in the supernatant.

**Table 4:** Protein composition of BSG protein extracts obtained through different extraction techniques. The molecular weight distribution and the associated protein types are listed in the order of their decreasing contribution to the total protein content.

Extraction Technique	Molecular Weight Distribution (kDa)		Associated Protein Type, if Known	References
Alcohol extract	55–80		C-hordeins	[16]
	35–50		B-hordeins	
Alkaline/acid	Light	Dark		[37]
	>10 (72%)	<5 (88%)		
	< 5 (21%)			
Alkaline/acid + ultrasound)	34–55		B-hordeins	[42]
	17		A-hordein	
	72		C-hordein	
	43		protein Z	
	35–50		B-hordein (31%)	[41]
	55–85		C-hordein (10%)	
	<20		γ-hordein (14%)	
	n.d.		β-amylase (19%)	
			Others (26%)	
Pressurized solvent extraction	35–50		B-hordein (45%)	[41]
	55–85		C-hordein (11%)	
	<20		γ-hordein (18%)	
			Others (26%)	
Pulsed electric field	Light	Dark	n.d.	[57]
	13.7	18.5		
		247		
Enzymatic (with or *w*/*o* ultrasound)	<15		n.d.	[53,54]
	>250			
Enzymatic + micro and nanofiltration	2–4.5 kDa		n.d.	[43]

n.d., no data

**Table 5:** Overview of selected studies that investigated physicochemical and functional properties of brewer’s spent grain proteins.

Source	Extraction	Treatment	Findings	Reference
Solubility				
Barley	Alkaline	Hydrolysis with Alcalase, Flavourzyme, Pepsin	Highest solubility increase in the range of pH 2–10 for Pepsin	[36]
Barley	Alkaline	Hydrolysis with Alcalase, Corolase, Flavourzyme, Promod	Alcalase achieved highest solubility increase with minimum solubility of around 45% at pH 4	[71]
Not Specified	Alkaline	Hydrolysis with Protamex, Flavourzyme	Highest amount of solubilized protein by combination of Protamex and Flavourzyme	[48]
Not Specified	Ethanolic−alkali mixture	*Rhizopus oligosporus* ATCC 64,063 fermentation	Solubility increases except for at pH4 for fermented BSG	[72]
Not Specified	Ultrasound-assisted enzymatic	Hydrolysis with Alcalase	Solubility >75% in pH 1–11, higher after ultrasound	[54]
Surface hydrophobicity (H_0_)				
Not Specified	Alkaline	none	H_0_ increased with extraction pH (8 < 11 < 12) and temperature (60 < 80 °C)	[73]
Barley and Rice	Enzymatic	Starch hydrolysis with glucoamylase followed by protein hydrolysis by alkaline protease	Could not be determined due to increased amount of small molecular weight peptides (<15 kDa)	[43,74]
Water absorption capacity				
Not Specified	Alkaline	none	3.2–5 g/g, no effect of extraction temperature (40 to 80 °C), increase with extraction pH from 8 to 12	[73]
Not specified	Ultrasound-assisted alkaline	none	3.7–5.5 g/g, increased with ultrasonic power but varied with ultrasonic time and duty cycle	[42]
Oil absorption capacity				
Not Specified	Alkaline	none	3.2–5.1 g/g, no effect of varied pH and temperature during extraction	[73]
Barley and Rice	Enzymatic	Starch hydrolysis with glucoamylase followed by protein hydrolysis by alkaline protease	1.82 ± 0.02 g/g	[43,74]
Not specified	Ultrasound-assisted alkaline	none	2.5–3.6 g/g, increased with ultrasonic power and extraction time	[42]
Emulsifying				
Not specified	Ultrasound-assisted alkaline	none	The ultrasound-treated extract has higher EAI and ESI	[42]
50% wheat/50% barley	Solvent extraction (Osborne)	none	Prolamins showed higher EAI and ESI compared to non-prolamins, particle size 6–7 μm	[75]
Barley	Alkaline	Hydrolysis with Alcalase, Flavourzyme, Pepsin	Highest EAI with Flavourzyme and Pepsin, highest ESI with Pepsin	[36]
Not Specified	Ethanolic−alkali mixture	*Rhizopus oligosporus* ATCC 64,063 fermentation of BSG	Fungi hydrolysis increases EAI and ESI (depending on pH)	[72]
Not specified	Alkaline	Alcalase hydrolysis	Hydrolyzed proteins showed higher EAI and ESI	[76]
Barley	Alkaline	Hydrolysis with Alcalase, Corolase, Flavourzyme, Promod	Corolase increases EAI at neutral pH, and ESI from pH 5–12	[71]
Not specified	Alkaline−enzymatic assisted	None	Increase in EAI after hydrolysis (protamex, flavourzyme), decreased ESI	[62]
Foaming				
Barley	Alkaline	Hydrolysis with Alcalase, Flavourzyme, Pepsin	Alcalase (DH 6%) highest foamability, Flavourzyme (DH 2%) highest foam stability	[36]
Not specified	Alkaline	None	Electrostatic complexation with carrageenan (iota/lambda) increases foamability and foam stability	[77]
Not specified	Ultrasound-assisted alkaline	none	The ultrasound-treated extract has higher foamability and stability	[42]
Not specified	Alkaline−enzymatic assisted	None	Increase in foamability and stability after hydrolysis (protamex, flavourzyme)	[62]
Barley	Alkaline	Hydrolysis with Alcalase, Corolase, Flavourzyme, Promod	No foam pH < 8, highest foamability and stability for unhydrolyzed protein extract at pH 12	[71]
Not specified	Alkaline	Alcalase hydrolysis	No significant difference in foamability and foam stability between hydrolyzed and control sample	[76]
Not Specified	Ethanolic−alkali mixture	*Rhizopus oligosporus* ATCC 64,063 fermentation of BSG	Higher foamability and foam stability for fungi hydrolyzed BSG	[72]
Gelling				
Not specified	Alkaline−enzymatic (carbohydrases) assisted	none	Gelation induced by acidification to pH 4.2, higher elasticity and yield stress for heat-treated protein extract	[78]
Barley and Rice	Enzymatic	Starch hydrolysis with glucoamylase followed by protein hydrolysis by alkaline protease	No gelation	[74]

## Data Availability

Not applicable.
